# PopSc: Computing Toolkit for Basic Statistics of Molecular Population Genetics Simultaneously Implemented in Web-Based Calculator, Python and R

**DOI:** 10.1371/journal.pone.0165434

**Published:** 2016-10-28

**Authors:** Shi-Yi Chen, Feilong Deng, Ying Huang, Cao Li, Linhai Liu, Xianbo Jia, Song-Jia Lai

**Affiliations:** 1 Farm Animal Genetic Resources Exploration and Innovation Key Laboratory of Sichuan Province, Sichuan Agricultural University, Chengdu, 611130, China; 2 College of Veterinary Medicine, Sichuan Agricultural University, Chengdu, 611130, China; National Cheng Kung University, TAIWAN

## Abstract

Although various computer tools have been elaborately developed to calculate a series of statistics in molecular population genetics for both small- and large-scale DNA data, there is no efficient and easy-to-use toolkit available yet for exclusively focusing on the steps of mathematical calculation. Here, we present PopSc, a bioinformatic toolkit for calculating 45 basic statistics in molecular population genetics, which could be categorized into three classes, including (i) genetic diversity of DNA sequences, (ii) statistical tests for neutral evolution, and (iii) measures of genetic differentiation among populations. In contrast to the existing computer tools, PopSc was designed to directly accept the intermediate metadata, such as allele frequencies, rather than the raw DNA sequences or genotyping results. PopSc is first implemented as the web-based calculator with user-friendly interface, which greatly facilitates the teaching of population genetics in class and also promotes the convenient and straightforward calculation of statistics in research. Additionally, we also provide the Python library and R package of PopSc, which can be flexibly integrated into other advanced bioinformatic packages of population genetics analysis.

## Introduction

Theoretical framework of population genetics had been significantly promoted due to the accumulated evidence in molecular biology, which resulted in an interdisciplinary Molecular Population Genetics more than two decades ago [1]. A primary task of molecular population genetics is to investigate the distribution and dynamic change of allele frequencies at population level in relation to a series of evolutionary processes and demographic events. Accordingly, both various mathematical models and a large number of statistical tests have been developed to comprehensively address these issues. A widely known example is that Japanese scientist Fumio Tajima proposed a simple and sophisticated statistic of Tajima *D* value for testing hypothesis of neutral evolution on basis of the observed DNA polymorphisms in 1989 [2], which has still been actively cited in enormous publications [3].

Prior to the advent of high-throughput sequencing techniques, the common molecular data available for studies of population genetics always only involve a short DNA fragment directly sequenced with tens of thousands of base pairs in length or several hundreds of polymorphism loci individually genotyped. By aiming at these small-scale and standard molecular data, kinds of computer programs, such as the actively cited DnaSP [4] and Arlequin [5], had been elaborately explored for calculating a number of statistics in population genetics with different strengths and weaknesses [6]. Recently, to better address the large-scale molecular data, such as genome-wide polymorphisms by massive resequencing, many high-throughput bioinformatic packages have been proposed for much more efficiently calculating the basic statistics, especially written in R programming language [7–9].

Despite the fact that there are various tools available for population genetics analysis on both small- and large-scale molecular data, we also found two limitations in this field deserving to be addressed. First, it is very inconvenient for using these existing tools, because all of them require strict format of input data, when you intend to compute certain statistics directly starting with the ready-made metadata of variants (such as allele frequencies) instead of raw DNA sequences. So, we believe that the easy-to-use online calculator would be very helpful for conducting some trivial tasks in teaching as well as research of molecular population genetics. Second, there is no toolkit available yet for exclusively performing the calculation step of statistics, which, in expectation, should be able to be directly and flexibly employed and incorporated into an advanced bioinformatic package. Such toolkit, therefore, could facilitate the development of analysis packages for bioinformatists without good background in population genetics, and also for biologists only holding the elementary skills in bioinformatics. In the present study, we specially addressed the two issues.

## Design of PopSc and Statistics

To address the issues as mentioned above, PopSc was designed to exclusively perform the calculation step of statistics of interest in molecular population genetics. Therefore, the initial input data into PopSc are these descriptive metadata, such as the allele or haplotype frequencies, number of segregating sites, counts of different mutation types, and mismatch distribution, etc., which must be generated from upstream raw DNA sequences or genotyping data in advance. After completing the computational duties, PopSc will directly pass the numerical output of statistic on to users. Such philosophy of design will promote the convenient calculation without requiring tedious input of raw DNA sequences and also guarantee the possibility of direct integration of PopSc toolkit into other advanced packages of bioinformatic analysis.

Generally guided by the popularity in scientific literatures, we comprehensively collected a total of 45 basic statistics in molecular population genetics into PopSc (Table 1). All of them could be roughly categorized into three classes, including (i) genetic diversity of DNA sequences, (ii) statistical tests for neutral evolution, and (iii) measures of genetic differentiation among populations. The required input data of PopSc for computing these statistics would be slightly different dependent on which one is selected, and each of them was clearly defined in the reference manuals. To facilitate practical uses, the calculation of each statistic is performed by one independent function. The mathematical formulas of these statistics were employed in intact from initial publications, meanwhile, all calculated results of PopSc were also carefully checked by either referring to values as being outputted by prevalent tools, such as DnaSP [4], or according to artificial verification step by step.

**Table 1:** Summaries of the included statistics into PopSc.

Classes	Statistics
**Genetic diversity**: the basic statistics about genetic diversity among a set of nucleotide sequences.	Heterozygosity *H*, Haplotype diversity *Hd* [10]; Nucleotide diversity *π* [11]; Average nucleotide differences *k* [12]; Polymorphism information content, *PIC* [13]
**Neutrality test**: the classical statistical tests for DNA neutral evolution based on the mutation frequencies, haplotype distribution, and mismatch distribution, respectively.	Tajima’s *D* [2]; Fu and Li’s *D*, *F*, D*, F* [14]; Strobeck’s *S*, Fu's *W*, *F*_s_, Watterson’s *W* [15, 16]; Fay and Wu's *H*, *H*_*n*_, Zeng's *E* [17, 18]; Ramos-Onsins’s *R*_2_, *R*_3_, *R*_4_, *R*_2*E*_, *R*_3*E*_, *R*_*4E*_, *Ch*, *Che*, *ku* [19]; Raggedness index *rg* [20]; Kelly’s *Z*_*ns*_, *Z*_*A*_ [21, 22]
**Population structure**: the statistics for measuring genetic differentiation among populations.	Wright's *F*_*ST*_, ${\bar{F}}_{ST}$, *F*_*IS*_ [23]; Nei's *G*_*ST*_, *D*_*ST*_, *J*_*T*_, *J*_*S*_, *R*_*ST*_ [24]; Hedrick $G_{ST}^{\prime }$ [25]; Jost *D* [26]; Weir and Cockerham’s *θ*_*U*_, *θ*_*RH*_, *θ*_*w*_, *f*_*U*_, *f*_*RH*_, *f*_*w*_ [27]

## Implementation and Availability

**Fig 1:**
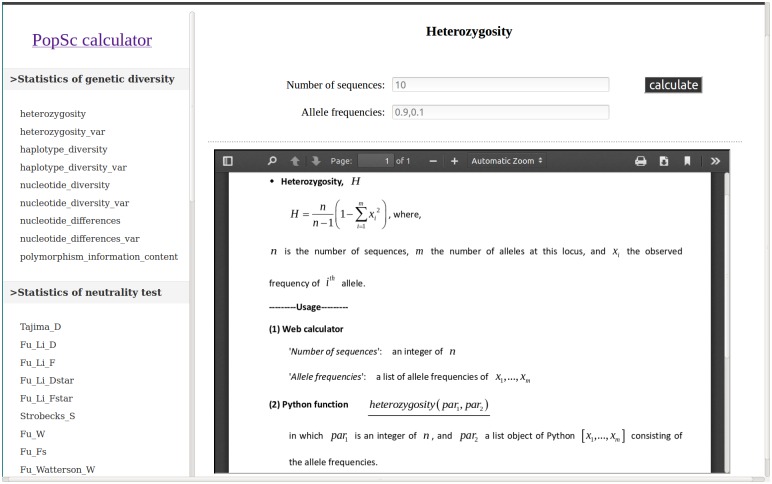
Screenshot of PopSc online calculator.

Because the web browser is independent of operating system and requires no installation, PopSc was first implemented as online calculator (Fig 1). The web-based calculator of PopSc provides the convenient and straightforward calculation of statistics without requiring *ab initio* input of DNA sequences or other types of raw molecular data. Mathematical formulas and the related documentation for each statistic are also clearly shown on the respective web pages, which would be very helpful for teaching the molecular population genetics in class; and such teaching purpose had also been successfully addressed by the very popular tool of GENALEX, which is an add-in program for Microsoft Excel [28].

During the past years, programming languages of Python and R are becoming more and more popular in bioinformatic analyses. Therefore, PopSc was further independently implemented and provided as the Python library and R package, which can be easily integrated into other advanced bioinformatic packages in molecular population genetics. The Python library and R package of PopSc are deposited in official repositories (http://pypi.python.org and http://cran.r-project.org, respectively) and could be installed by standard commands. The web-based calculator, source codes and reference manuals of PopSc could also be freely available at: http://chenshiyi.com/popsc.html.

Of course, PopSc is not an upgrade or even a replacer to the exiting computer tools because it just performs the step of mathematical calculation for each statistic. Therefore, PopSc only accepts the descriptive metadata, which must be independently prepared from raw DNA sequences or genotyping data with the aid of the custom scripts or other computer tools, such as MEGA [29]. PopSc certainly remains open for the additional implementation of other statistics in molecular population genetics.
